# Blockade of adenosine A_2A_ receptors prevents interleukin-1β-induced exacerbation of neuronal toxicity through a p38 mitogen-activated protein kinase pathway

**DOI:** 10.1186/1742-2094-9-204

**Published:** 2012-08-20

**Authors:** Ana Patrícia Simões, João A Duarte, Fabienne Agasse, Paula Margarida Canas, Angelo R Tomé, Paula Agostinho, Rodrigo A Cunha

**Affiliations:** 1 Center for Neurosciences of Coimbra, Institute of Biochemistry, Faculty of Medicine, University of Coimbra, 3004-504, Coimbra, Portugal; 2 Department of Life Sciences, Faculty of Sciences and Technology, University of Coimbra, 3301-401, Coimbra, Portugal

**Keywords:** Adenosine, A_2A_ receptor, Interleukin 1β, Neurodegeneration, p38 MAPK, Calcium

## Abstract

**Background and purpose:**

Blockade of adenosine A_2A_ receptors (A_2A_R) affords robust neuroprotection in a number of brain conditions, although the mechanisms are still unknown. A likely candidate mechanism for this neuroprotection is the control of neuroinflammation, which contributes to the amplification of neurodegeneration, mainly through the abnormal release of pro-inflammatory cytokines such as interleukin(IL)-1β. We investigated whether A_2A_R controls the signaling of IL-1β and its deleterious effects in cultured hippocampal neurons.

**Methods:**

Hippocampal neuronal cultures were treated with IL-1β and/or glutamate in the presence or absence of the selective A_2A_R antagonist, SCH58261 (50 nmol/l). The effect of SCH58261 on the IL-1β-induced phosphorylation of the mitogen-activated protein kinases (MAPKs) c-Jun N-terminal kinase (JNK) and p38 was evaluated by western blotting and immunocytochemistry. The effect of SCH58261 on glutamate-induced neurodegeneration in the presence or absence of IL-1β was evaluated by nucleic acid and by propidium iodide staining, and by lactate dehydrogenase assay. Finally, the effect of A_2A_R blockade on glutamate-induced intracellular calcium, in the presence or absence of IL-1β, was studied using single-cell calcium imaging.

**Results:**

IL-1β (10 to 100 ng/ml) enhanced both JNK and p38 phosphorylation, and these effects were prevented by the IL-1 type 1 receptor antagonist IL-1Ra (5 μg/ml), in accordance with the neuronal localization of IL-1 type 1 receptors, including pre-synaptically and post-synaptically. At 100 ng/ml, IL-1β failed to affect neuronal viability but exacerbated the neurotoxicity induced by treatment with 100 μmol/l glutamate for 25 minutes (evaluated after 24 hours). It is likely that this resulted from the ability of IL-1β to enhance glutamate-induced calcium entry and late calcium deregulation, both of which were unaffected by IL-1β alone. The selective A_2A_R antagonist, SCH58261 (50 nmol/l), prevented both the IL-1β-induced phosphorylation of JNK and p38, as well as the IL-1β-induced deregulation of calcium and the consequent enhanced neurotoxicity, whereas it had no effect on glutamate actions.

**Conclusions:**

These results prompt the hypothesis that the neuroprotection afforded by A_2A_R blockade might result from this particular ability of A_2A_R to control IL-1β-induced exacerbation of excitotoxic neuronal damage, through the control of MAPK activation and late calcium deregulation.

## Introduction

Neuroinflammation is a common feature of most neurological disorders and pathological conditions in the brain, involving recruitment of microglia cells and release of a large number of inflammatory mediators, including pro-inflammatory cytokines [1,2]. One of the most prominent pro-inflammatory cytokines is interleukin (IL)-1β, which is usually present at low levels in the healthy brain, and modulates several physiological functions, including synaptic plasticity phenomena [3,4]. However, higher levels of IL-1β (as typically found after acute and chronic brain insults) inhibit synaptic plasticity, which is considered to be linked with the depression of brain function associated with inflammatory conditions [5].

In addition to modulating synaptic plasticity, IL-1β primes neurons to undergo excitotoxic death, an effect that probably results from a direct neuronal action, as gauged by the parallel *in vivo* and *in vitro* effects of IL-1β [6-8]. This effect has been related to the ability of IL-1β to recruit various members of the mitogen-activated protein kinase (MAPK) pathway [9,10] that are known to control neurodegeneration [11,12], and to the ability of IL-1β to potentiate responses mediated by glutamate receptors of the N-methyl-D-aspartic acid (NMDA) subtype [7,13,14], key players in neurodegeneration [15].

We previously put forward the concept that adenosine A_2A_ receptors (A_2A_R) control synaptic plasticity [16] and neurodegeneration [17,18]. The combined observations that neuroinflammatory conditions and IL-1β trigger purine release [19,20], and that their action through A_2A_R activation is involved in inflammation-associated damage [8,21], indicates that A_2A_R tightly controls neuroinflammation, as it does in the case of peripheral inflammation [22]. We and others have previously shown that A_2A_R control the recruitment of microglia [23,24] and the production of pro-inflammatory mediators, including IL-1β [21,25]. However, because A_2A_R also control the direct effects on neurons of a number of deleterious stimuli such as the apoptotic inducer, staurosporine [26] or the Alzheimer’s disease-related peptide, β-amyloid [27], we investigated whether A_2A_R could also control the effects of IL-1β on neurons. We chose to test this possibility in hippocampal neurons because the hippocampus displays high levels of IL-1β and its receptor, and because the physiopathological effects of IL-1β in this brain region are well-characterized [28].

## Methods

### Ethics approval

All experiments were approved by the Ethics committee of the Center for Neurosciences and Cell Biology, Faculty of Medicine, University of Coimbra. All animals used in the study were handled in accordance with EU guidelines (86/609/EEC).

### Animals

Male Wistar rats (Charles River, Barcelona, Spain) aged 8 weeks old, were used for total, synaptic and sub-synaptic membrane preparations. Rats were maintained in the animal facilities and handled only at the time of sacrifice, always at the same hour of the day because there is circadian regulation of IL-1β levels in the brain [30]. Rats were deeply anesthetized with halothane before being killed by decapitation. Total and synaptic membranes were prepared from the same group of animals and another group of rats was used for preparing sub-synaptic membranes.

Embryos from 2 to 4 months old female Wistar rats were used for the primary neuronal cultures. Pregnant females were anaesthetized with halothane on the eighteenth day of pregnancy, and the embryos removed.

### Preparation of total membranes from the hippocampus

The purification of total membranes from the rat hippocampus was performed essentially as described previously [29]. After removal of the brain, the hippocampi were isolated and homogenized in a sucrose solution (0.32 mol/l sucrose containing 1 mmol/l EDTA, 10 mmol/l HEPES and 1 mg/ml BSA; pH 7.4) at 4 °C. This homogenate was separated by centrifugation at 3,000 *g* for 10 minutes at 4°C. The supernatant was removed and again separated by centrifugation at 100,000 *g* for 30 minutes at 4°C. The obtained pellets contained the total cytoplasmic membranes and were resuspended in 5% SDS with 0.1 mmol/l of PMSF and finally, after determination of protein density using the bicinchoninic acid method, diluted in SDS-PAGE buffer (6% of a Tris.Cl/SDS solution (0.5 mol/l Tris and 0.4% SDS, pH 6.8 corrected with HCl and filtered with 0.45 μm pore filters), 30% glycerol, 10% SDS, 0.6 mol/l DTT and 0.012% of bromophenol blue) and boiled at 95 °C during 5 minutes for western blotting analysis.

### Preparation of hippocampal synaptosomes

The preparation of hippocampal synaptosomes from rats was carried out essentially as described previously [29]. After removal of the brain, the dissected hippocampi were homogenized in the same sucrose solution described above, and the homogenates were separated by centrifugation at 3,000 *g* for 10 minutes at 4°C. The supernatant was removed and again separated by centrifugation at 14,000 *g* for 12 minutes at 4°C. The resulting pellet (the P2 fraction) was resuspended in 1 ml of a 45% (v/v) Percoll solution prepared in a Krebs-HEPES-Ringer (KHR) solution (140 mmol/l NaCl, 1 mmol/l EDTA, 10 mmol/l HEPES, 5 mmol/l KCl, 5 mmol/l glucose; pH 7.4) at 4°C. This homogenate was then separated by centrifugation at 12,650 *g* for 2 minutes at 4°C in an Eppendorf microcentrifuge. The resulting white top layer (synaptosomal fraction) was collected, resuspended in 1 ml of KHR, and separated by centrifugation at 12,650 *g* for 2 minutes at 4°C. The pellets (synaptosomes) were resuspended and diluted in the same solutions as total cytoplasmic membranes for western blotting analysis.

### Preparation of sub-synaptic membrane fractions

To gauge the sub-synaptic localization of IL-1β receptors, we used purification of sub-synaptic membrane fractions, following the method initially published by Phillis *et al*. [31] and adapted by our group [29]. This method can separate, with over 90% efficiency, membrane proteins from the active zone (enriched in synaptosomal-associated protein (SNAP)-25), the cytoskeletal post-synaptic density (PSD) (enriched in the protein PSD-95), and the non-active zone fraction (enriched in synaptophysin).

This sub-synaptic fractionation begins with the purification of synaptosomes. For this purpose, the hippocampi were homogenized in 2.5 ml of isolation buffer (0.32 mol/l sucrose, 0.1 mmol/l CaCl_2_, 1 mmol/l MgCl_2_, 1 mmol/l phenylmethanesulfonylfluoride (PMSF), and 1 μg/ml of a protease inhibitor cocktail (10 μg/ml of chymostatin, leupeptin, antipain and pepstatin A), and 100 μl of this mixture was stored at −80°C for later analysis. The homogenate was transferred to 50 ml centrifuge tubes at 4°C, and 12 ml of a 2 mol/l sucrose solution was added, together with 5 ml of 0.1 mmol/l CaCl_2_ to yield a final solution with 1.25 mol/l sucrose. This was then divided into two tubes (Ultra-Clear^TM^; Beckman Coulter Inc., Brea, CA, USA) and 2.5 ml of 1 mol/l sucrose solution (containing 0.1 mmol/l CaCl_2_) was carefully layered over the solution in each tube. The tubes were equilibrated with isolation buffer and separated by centrifugation at 100,000 *g* for 3 hours at 4°C. The synaptosomes were captured at the interface between the 1.25 mol/l and 1 mol/l sucrose solutions, and were then diluted 1:10 in isolation buffer. After centrifugation at 15,000 *g* for 30 minutes at 4°C the pellet was resuspended in 1.1 ml isolation buffer, and 100 μl of this mixture was stored at −80°C for later analysis.

For preparation of the various sub-synaptic fractions, the synaptosomes were diluted in 10 ml of a cooled solution of 0.1 mmol/l CaCl_2_ in 50 ml beakers, and with 10 ml of the solubilization buffer pH 6.0 (40 mmol/l Tris, 2% Triton X-100, pH 6.0 precisely adjusted to pH 6.0 at 4°C) . The mixture was gently stirred for 30 minutes on ice, and divided between two tubes (Ultra-Clear^TM^; Beckman Coulter Inc.), which were then spun at 40,000*g* for 30 minutes at 4°C in a centrifuge. The pellet contained the synaptic fractions and the supernatant the extra-synaptic proteins. The supernatants were kept on ice, and the pellet was resuspended in 5 ml of solubilization buffer (20 mmol/l Tris with 1% Triton X-100), precisely adjusted to pH 8.0 at 4°C. This mixture was gently stirred for 30 minutes on ice, and separated by centrifugation at 40,000 *g* for 30 minutes at 4°C. The pellet contained the PSD and the supernatant contained the pre-synaptic proteins. The supernatant was transferred to centrifuge tubes, and the pellet resuspended in 5 ml of the solubilization buffer (pH 8.0) and again stirred gently for 30 minutes on ice, followed by further centrifugation at 40,000*g* for 30 minutes at 4°C. The supernatant was added to the pre-synaptic fraction, and the pellet, containing the re-extracted post-synaptic fraction, was resuspended in a minimal volume of 5% SDS solution with 0.1 mmol/l PMSF for subsequent western blotting analysis. To concentrate the extra-synaptic and pre-synaptic proteins, a volume of 40 ml of cold acetone (−18°C) was added to each 10 ml of the supernatants and kept overnight at −20°C. Both fractions were pelleted by centrifugation at 18,000 *g* for 30 minutes at −15°C, then both pellets were resuspended in circa 50 μl of 5% SDS with 0.1 mmol/l PMSF for subsequent western blotting analysis.

### Preparation of primary neuronal cultures

Rat hippocampal neuronal cultures were prepared essentially as described previously [27]. Embryos were removed by cesarean section, and placed in a container with sterile Hank’s balanced salt solution (HBSS) pH 7.2 without calcium and magnesium (137 mmol/l NaCl, 5.36 mmol/l KCl, 0.44 mmol/l KH_2_PO_4_, 4.16 mmol/l NaHCO_3_, 0.34 mmol/l Na_2_HPO_4_, 5 mmol/l glucose, with the addition of 0.001% phenol red), which was sterilized by filtration though a 0.2 μm filter. The hippocampi of the embryos were dissected in the HBSS solution and digested with 2 mg/ml trypsin (from bovine pancreas; Sigma-Aldrich, Sintra, Portugal) for 10 minutes at 37°C in a water bath. The trypsin reaction was stopped with 1.5 mg/ml of trypsin inhibitor (from bovine pancreas; Sigma-Aldrich), and the hippocampi were washed once with HBSS. The HBSS was carefully removed and 1 ml of the Neurobasal medium (Gibco/Invitrogen, Lisboa, Portugal), supplemented with a 1:50 dilution of B27, 0.5 mg/ml L-glutamine, 25 μmol/l L-glutamate and antibiotics (penicillin and streptomycin; 1:100; Gibco/Invitrogen), was added. The tissue was further mechanically dissociated using a 1 ml micropipette until it formed a homogeneous mass.

The cells were counted using a hemocytometer under a light microscope. Further dilutions were made using the supplemented Neurobasal medium until the final desired cell density was reached. Neurons were then plated onto plates and coverslips that were coated with poly-D-lysine (0.1 mg/ml prepared in 166 mmol/l borate buffer; pH 8.2). For immunocytochemistry and viability assays, cells were plated onto 16-mm diameter coverslips in 12-well dishes at a density of 50,000 cells/coverslip, for single-cell calcium image, they were plated onto 12-mm diameter coverslips at a density of 37,000 cells/coverslip, and for western blotting assays, they were plated onto six-well dishes at a density of 800,000 cells/well. In all cases, they were incubated for 7 days *in vitro* in a humidified atmosphere of 95% O_2_/5% CO_2_ in an incubator kept at 37°C.

Using immunocytochemical identification of an astrocytic marker (glial fibrillary acidic protein) and two neuronal markers (microtubule-associated protein-2 and β-tubulin III), we verified that all neuronal cultures were >98% pure.

### Drug exposure of cultured neurons

In this study, we addressed two fundamentally different questions, using different protocols. First, we assessed whether exposure to IL-1β affected intracellular biochemical markers, including the activation (that is, phosphorylation) of different MAPKs. This was carried out by incubating cultured neurons for various periods (5 to 180 minutes) with various concentrations of IL-1β (1 to 10 ng/ml; R&D Systems, Minneapolis, MN, USA). Afterwards, the cell medium was aspirated, and the cells were either lysed for western blotting analysis or fixed for immunocytochemistry analysis (see below). We tested the ability of the adenosine A_2A_R antagonist, SCH58261 (2-(2-furanyl)-7-(2-phenylethyl)-7H-pyrazolo[4,3-e][1,2,4] triazolo[1,5-c]pyrimidin-5-amine) to modify IL-1β-induced phosphorylation of different MAPKs. We added 50 nmol/l SCH58261 to the cellular medium (generous gift of S. Weiss, Vernalis, UK) 20 minutes before the addition of IL-1β, and it remained in the solution throughout the protocol. We selected this antagonist in view of our previous validation of its selectivity and efficiency [27,32].

The second question related to the ability of IL-1β to control glutamate-induced neurotoxicity. Cultured neurons were exposed to 100 ng/ml IL-1β for 5 minutes before exposure to either vehicle (Krebs buffer: 150 mmol/l NaCl, 5 mmol/l KCl, 10 mmol/l glucose, 10 mmol/l HEPES, 2 mmol/l CaCl_2_ and 1 mmol/l MgCl_2_, pH 7.4) or 100 μmol/l L-glutamate (Sigma-Aldrich) for 25 minutes. The neurons were then washed three times with Krebs buffer, then Neurobasal medium was added, and the neurons were incubated for 24 hours until we carried out analysis of neuronal dysfunction or damage (see below). To test the ability of 50 nmol/l SCH58261 to modify glutamate-induced neurotoxicity, SCH58261 was added 20 minutes before glutamate, and remained in all solutions until we carried out analysis of neuronal dysfunction or damage. Likewise, when we tested the ability of an inhibitor of the mitogen-activated protein kinase p38 (SB 203580; 10 μmol/l) or of a JNK inhibitor (SP600125; 10 μmol/l) (both Ascent Scientific, Bristol, UK) to modify glutamate-induced neurotoxicity, each of these inhibitors was added 30 to 40 minutes before glutamate, and was present in all solutions until we carried out analysis of neuronal dysfunction or damage.

### Western blotting analysis

For western blotting analysis, membranes (total or from synaptosomes and sub-synaptic fractions) were resuspended in a 5% SDS solution with 0.1 mmol/l PMSF. The cultured neurons were lysed in radio-immunoprecipitation assay (RIPA) buffer (50 mmol/l Tris pH 7.4, 150 mmol/l NaCl, 1% Nonidet P-40, 0.5% Na-deoxycholate, 1 mmol/l EDTA and 0.1% SDS, supplemented with 1 mmol/l PMSF, 1 μg/ml protease inhibitor cocktail (chymostatin, leupeptin, antipain and pepstatin; CLAP), 1 mmol/l dithiothreitol, 1 mmol/l sodium orthovanadate and 1 mmol/l sodium fluoride). Protein quantification in all these samples was performed using the bicinchoninic acid method.

The samples diluted in SDS-PAGE buffer and the pre-stained molecular weight markers (dual-color standards; BioRad, Amadora, Portugal) were loaded and separated by SDS-PAGE electrophoresis (in 7.5% polyacrylamide resolving gels with 4% polyacrylamide stacking gels, both gels 1.5 mm thick) under denaturating/reducing conditions, using a bicine-buffered solution (20 mmol/l Tris, 192 mmol/l bicine, and 0.1% SDS; pH 8.3) at 80 to 100 mV. After separation through electrophoresis, the proteins were transferred from the gel (applying a current of 1 A for 1.5 hours at 4°C under constant agitation) to polyvinylidene difluoride (PVDF) membranes (0.45 μm pore diameter; GE Healthcare, Little Chalfont, Buckinghamshire, UK), previously activated (for 5 to 15 seconds) in 100% methanol, hydrated for 5 minutes in distilled water, and equilibrated for 30 minutes using a 3-(cyclohexylamino)-1-propane-sulfonic acid (CAPS)-buffered solution with methanol (10 mmol/l CAPS and 10% (v/v) methanol; pH 11). Membranes were then blocked for 1 hour at room temperature with 5% BSA in Tris-buffered saline (20 mmol/l Tris, 140 mmol/l NaCl; pH 7.6) with 0.1% Tween 20 added (TBS-T). Membranes were then incubated overnight at 4°C with the primary antibodies diluted in TBS-T with 5% BSA. After being washed three times for 15 minutes each with TBS-T, the membranes were incubated for 1 hour at room temperature with the phosphatase-linked secondary antibodies, also diluted in TBS-T with 5% BSA. Again, membranes were washed three times for 15 minutes each with TBS-T, and then incubated with enhanced chemifluorescence substrate (ECF; GE Healthcare) for varying times, up to a maximum of 5 minutes. Finally, proteins were detected and analyzed (Molecular Imager VersaDoc 3000 and Quantity One software; both BioRad).

Reprobing of the same membranes with the different antibodies was then performed. The ECF was removed by washing in 40% methanol for 30 minutes, and the previous antibodies were removed in a mild stripping solution (0.2 mol/l glycine with 0.1% SDS and 1% (v/v) Tween 20; pH 2.2) for 1 hour. After washing three times for 20 minutes each with TBS-T, membranes were again blocked with TBS-T with 5% BSA before incubation first with the new primary antibody and next with the appropriate secondary antibody.

The following primary antibodies were used: mouse anti-phospho-p38 MAPK, rabbit anti-p38 MAPK, mouse anti-phospho-stress-activated protein kinase (SAPK)/JNK, rabbit anti-SAPK/JNK, mouse anti-phospho-p44/p42 MAPK, extracellular signal-regulated kinase (ERK)1/2 and rabbit anti-p44/p42 MAPK (all at 1:1000 all from Cell Signaling Technology, Beverly, MA, USA), rabbit anti-IL-1β receptor 1 (1:200; Santa Cruz Biotechnology, Santa Cruz, CA, USA), mouse monoclonal anti-SNAP25 (1:5000), mouse anti-PSD95 (1:20,000) and mouse anti-synaptophysin (1:20,000) (all from Sigma-Aldrich, St Louis, MO, USA). The following secondary antibodies were also used: goat anti-rabbit IgG antibody conjugated with alkaline phosphatase (1:20,000; Amersham Biosciences Inc., Piscataway, NJ, USA) and goat anti-mouse IgG antibody conjugated with alkaline phosphatase (1:10,000; Santa Cruz Biotechnology).

### Immunocytochemistry analysis

Immunocytochemistry in hippocampal neuronal cultures was carried out essentially as described previously [26,27] to evaluate the localization in neurons of the activated/phosphorylated forms of the MAPKs JNK and p38, induced by the pro-inflammatory cytokine IL-1β. After an incubation period of 15 minutes with 100 ng/ml IL-1β, the cells were rapidly washed first with Neurobasal medium then with PBS (140 mmol/l NaCl, 3 mmol/l KCl, 26 mmol/l NaH_2_PO_4_ and 15 mmol/l KH_2_PO_4_; pH 7.4). Cells were then fixed with 4% paraformaldehyde (prepared in 0.9% NaCl with 4% sucrose) for 30 minutes, washed three times (for 1 minute each) with PBS, permeabilized with PBS containing 0.2% Triton X-100 for 5 minutes, washed twice with PBS, and incubated in PBS containing 3% BSA for 1 hour at room temperature to block nonspecific binding of antibodies. Cells were then incubated overnight at 4°C with primary antibodies prepared in PBS plus 3% BSA, washed three times with PBS, and then incubated for 1 hour at room temperature with the appropriate fluorophore-conjugated secondary antibodies. The primary antibodies tested were either mouse anti-phospho-p38 MAPK or mouse anti-phospho-SAPK/JNK antibodies as above (both 1:100; Cell Signaling Technologies) and the secondary antibody was a donkey anti-mouse IgG antibody coupled with Alexa Fluor 488 (1:200; Invitrogen). Mounting medium (H-1500; Vectashield) with the nucleus-specific fluorescent marker 4′,6-diamidino-2-phenylindole (DAPI) (both Vector Laboratories, Cambridgeshire, UK), was used to maintain fluorescence. Finally, the preparations were examined by transmission and fluorescence microscopy (either a Zeiss Axiovert 200 equipped with AxioVision software (version 4.6) or a Zeiss LSM 510Meta laser scanning confocal microscope; all PG-Hitec, Lisbon, Portugal).

### Evaluation of dysfunction and damage of cultured neurons

There is controversy regarding the quantification of neuronal viability, because all available methods display accuracy problems, which depend on the experimental conditions [33]. Therefore, we decided to use two different methods previously used by our group [26,27] to assess the effect of a short exposure to glutamate on neuronal viability, dysfunction, and/or damage, namely staining with propidium iodide (PI) and SYTO-13 (to gauge the degree of necrosis and primary and secondary apoptosis), and assessment of lactate dehydrogenase (LDH) release (to evaluate rupture of cell membrane).

### SYTO-13 and propidium iodide assay

SYTO-13 (Molecular Probes, Leiden, the Netherlands) is a cell-permeating nucleic-acid stain that increases its fluorescence upon binding to nucleic acids, thus, the pattern of SYTO-13 staining allows the visualization of viable cells (darker, homogeneous, and larger green fluorescent nuclei) and apoptotic cells in which the plasmatic membrane is still intact (brighter, fragmented, and smaller green fluorescent nuclei). PI (Molecular Probes) also binds nucleic acids, resulting in strong red fluorescent enhancement; however, because this dye cannot penetrate cytoplasmic membranes, it only stains cells with a damaged plasma membrane, that is, necrotic cells (darker, homogeneous, and larger red fluorescent nuclei) and cells undergoing secondary apoptosis (small, very bright red spots of fragmented nuclei). To determine neuronal damage, cultured neurons were washed three times with Krebs buffer, then incubated for 3 minutes with a mixture of SYTO-13 (4 μmol/l) and PI (4 μg/ml) prepared in Krebs buffer. After slides were coverslipped, neurons were visualized and counted using fluorescence microscopy (Zeiss Axiovert 200; PG-Hitec). At least six fields per coverslip were analyzed, counting a total of approximately 300 cells.

### Lactate dehydrogenase assay

LDH (EC 1.1.1.27) is a cytoplasmic oxidoreductase that catalyses the interconversion of pyruvate and lactate with concomitant interconversion of NADH and NAD^+^. Upon overt cell damage leading to a compromise of plasma-membrane integrity, LDH is released into the extracellular space. Being a fairly stable enzyme, it has been widely used to evaluate the degree of damage induced by insults to cells, especially in the context of cell death occurring mainly through necrosis. In this study, LDH activity was measured spectrophotometrically by assessing the rate of conversion of NADH to NAD^+^ using optical density at 340 nm. Thus, to determine neuronal damage, the medium (extracellular fraction) was aspirated and kept at 4°C until analysis. The plated neurons were lysed by three freeze/thaw cycles with 1 ml HEPES buffer (10 mmol/l; pH 7.4) containing 0.02% Triton X-100. The lysates (intracellular fraction) were also kept at 4°C for analysis. Before the assay, both intracellular and extracellular fractions were separated by centrifugation for 10 minutes at 14,000 rpm in a microcentrifuge (Eppendorf) at 4°C. The pellets (cellular organelles and membrane fractions) were discarded, and the supernatants were used to measure LDH activity as follows. Samples of these supernatants (100 μl) were diluted with 0.5 ml of Tris-NaCl buffer pH 7.2 (81.3 mmol/l Tris, 203.3 mmol/l NaCl with 9.76 mmol/l pyruvate) at 30°C. Reactions were started by adding 2.5 ml of 0.244 mmol/l NADH into the Tris-NaCl buffer solution. Absorbance was measured at 340 nm, and the decrease in absorbance was followed every 0.5 seconds for 2 minutes; the slope of the decrease showed the LDH activity. The percentage of LDH leakage was calculated using the ratio between extracellular LDH activity and the sum of intracellular and extracellular LDH activity, and results were expressed as percentage of control values. Determinations were performed in triplicate for each sample, and the results averaged.

### Single-cell calcium imaging

This was carried out essentially as described previously [34], using Fura-2-acetoxymethyl ester (Fura-2-AM), a membrane-permeable and calcium-sensitive radiometric dye, to fluorimetrically measure variations in the intracellular free calcium concentration ([Ca^2+^_i_) by monitoring its ratio of absorption at 510 nm upon excitation at 380 nm or 340 nm. Briefly, hippocampal neurons, plated onto coverslips, were loaded with 5 μmol/l Fura-2-Amol/l and 0.02% pluronic acid F-127 (both Molecular Probes, Leiden, Netherlands) for 30 minutes in Krebs buffer (150 mmol/l NaCl, 5 mmol/l KCl, 10 mmol/l glucose, 10 mmol/l HEPES, 2 mmol/l CaCl_2_ and 1 mmol/l MgCl_2_; pH 7.4) supplemented with 0.1% fatty-acid free BSA (Sigma-Aldrich), at 37°C in an incubator in a atmosphere of 95% CO_2_/5% O_2_. After washing three times with Krebs buffer to remove excess probe, coverslips were placed in a superfusion chamber (RC-20; Warner Instruments, Harvard, UK) on the stage of an inverted fluorescence microscope (Axiovert 200; Carl Zeiss, Jena, Germany). Hippocampal neurons were alternately excited at 340 and 380 nml using an optical splitter (Lambda DG4; Sutter Instruments, Novato, CA, USA) ,and the emitted fluorescence was captured through a 40× oil objective connected to a digital camera (CoolSNAP; Roper Scientific, Trenton, NJ, USA). Acquired images were processed using MetaFluor software (Universal Imaging Corp., Buckinghamshire, UK). The areas of the cell bodies were drawn, and the average value of pixel intensities was evaluated at each time point. Image acquisition was performed every second for a total of 35 minutes. Results were expressed by plotting the time course of the ratio of fluorescence intensity emitted at 510 nm (using a 510 nm band-pass filter; Carl Zeiss) after excitation (750 ms, using the Lambda splitter) alternately at 340 and 380 nm [34]. All of the compounds tested were prepared in Krebs buffer and added to the cultured neurons by superfusion using a rapid-pressurization system (AutoMate Scientific, Berkeley, CA, USA) in 95% O_2_/5% CO_2_). The basal ratio was measured for the first 2 minutes of the experiment, before the stimuli were made. When present, 100 ng/ml IL-1β was added for 5 minutes before the addition of 100 μmol/l glutamate, then the cells were incubated for a further 15 minutes, after which they were washed with Krebs buffer. To assure that the selected cell bodies belonged only to neurons, a challenge with 50 mmol/l KCl was carried out at the end of each experiment. When the A_2A_R antagonist (50 nmol/l SCH58261), the p38 inhibitor (10 μmol/l SB203580), or the JNK inhibitor (10 μmol/l SP600125) were tested, each of these drugs was incubated with the cells for 20 to 40 minutes before the beginning of the experiment, and was present throughout the experiment.

### Statistical analysis

Values are presented as mean ± SEM. Either Student’s *t*-test for independent means or a one-way analysis of variance (ANOVA) followed by Bonferroni analysis of variance, was used to define statistical differences between values, which were considered significant at *P* < 0.05 unless otherwise specified.

## Results

### Effect of interleukin-1β on neuronal MAPKs

Most cell functions regulated by pro-inflammatory cytokines such as IL-1β are triggered by cytokine-induced activation (that is*,* phosphorylation) of MAPKs, including ERK, JNK, and p38 [4,35]. Thus, we studied how exposure of rat hippocampal cultured neurons to IL-1β (1 to 100 ng/ml) affected the phosphorylation of various MAPKs.

**Figure 1:**
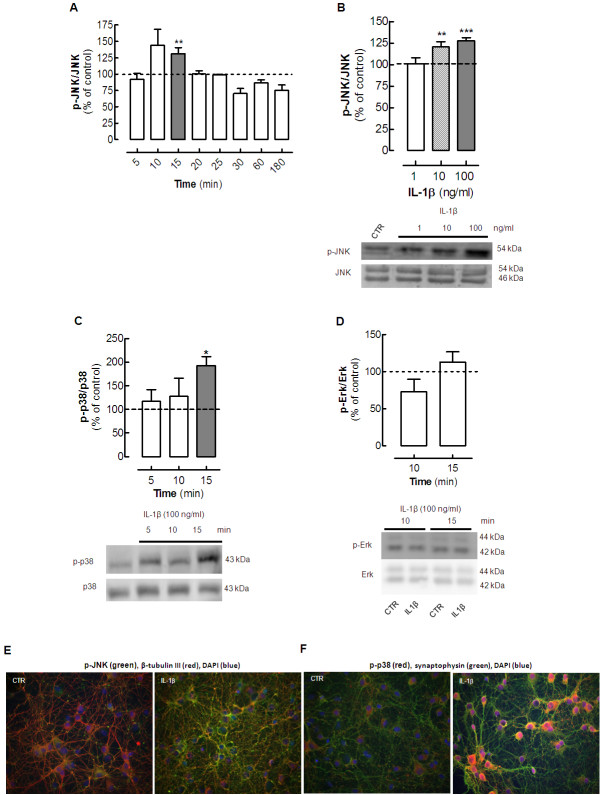
**Interleukin (IL)-1βtriggers thephosphorylation ofthe mitogen-activatedprotein kinases(MAPKs) Junkinase (JNK)and p38in hippocampalcultured neurons.** Rat hippocampal neurons at 7 days *in vitro* (DIV), were exposed to various concentrations of IL-1β (1 to 100 ng/ml, prepared in Krebs buffer; pH 7.4) for different times (5, 10, 15, 20, 25, 30, 60 and 180 minutes), after which cells were either lysed on ice in radio-immunoprecipitation assay (RIPA) buffer for (**A–D**) western blotting analysis or fixed for (**E–F**) immunocytochemical analysis of the pattern of activation (phosphorylation) of the various MAPKs. (**A**) IL-1β (10 ng/ml) activated JNK in a time-dependent manner, reaching significance at 10 to 15 minutes of incubation with IL-1β. (**B**) Activation of JNK increased in line with increasing concentrations of IL-1β. (**C**) After 15 minutes of exposure to 100 ng/ml IL-1β, p38 MAPK was also activated. (**D**) However, under similar experimental conditions, IL-1β failed to significantly affect the amount of phosphorylated p42 ERK. In each panel, the bar graphs display the mean ± SEM of four to eight cultures, and the blots below illustrate a representative experiment. Membranes were first used to detect the phosphorylated MAPK, and then reprobed for the total amount of MAPK. MAPK activation was calculated as a ratio between the phosphorylated and total immunoreactivities, expressed as a percentage of the control values (that is, in the absence of IL-1β). The values are mean ± SEM of four to eight experiments, *P < 0.05, **P < 0.01 and ***P < 0.001. (**E**) Representative immunocytochemistry images of hippocampal neurons (left) in the absence and (right) after 15 minutes in the presence of 100 ng/ml IL-1β, probed for the phosphorylated form of JNK (green fluorescence) which co-localized with the class III β-tubulin, a constitutive protein exclusively of neuronal microtubules (red fluorescence). (**F**) Representative immunocytochemistry images of hippocampal neurons (left) in the absence and (right) after 15 minutes in the presence of 100 ng/ml IL-1β, probed for the phosphorylated form of p38 (red fluorescence) which co-localized with synaptophysin (green fluorescence). (**E,F**) The neuronal nuclei were stained with DAPI (blue fluorescence).

We found that 10 ng/ml IL-1β rapidly activated JNK in cultured neurons in a transient manner, (Figure 1A), reaching significance only after 15 minutes of incubation (131.2 ± 9.4% of control; *P* < 0.01; n = 8;) and decreasing to basal levels thereafter until 3 hours of exposure. The activation of JNK also depended on the concentration of IL-1β, being significant at 10 and 100 ng/ml (*P* < 0.001, n = 8) (Figure 1B).

Because the highest concentration of IL-1β produced more robust results, we tested the effect of incubation for 5 to 15 minutes with 100 ng/ml IL-1β on the activation (phosphorylation) of p38. The phosphorylated p38 levels were significantly (*P* < 0.05) increased (192.8 ± 18.8%, n = 5) in cultured neurons after 15 minutes of incubation with IL-1β (Figure 1C). However, this concentration of IL-1β failed to activate ERK in hippocampal cultured neurons in the same period of incubation in which it activated both JNK and p38 MAPK (at 10 to 15 minutes of incubation) (Figure 1D).

Immunocytochemical analysis of hippocampal cultured neurons confirmed that exposure to 100 ng/ml IL-1β for 15 minutes triggered an evident increase of the immunoreactivity of phosphorylated JNK throughout the neurons (Figure 1E) and also of phosphorylated p38, mainly in neuronal cell bodies (Figure 1F).

### The effect of interleukin-1β on neuronal MAPKs is controlled by interleukin-1β type I receptors

**Figure 2:**
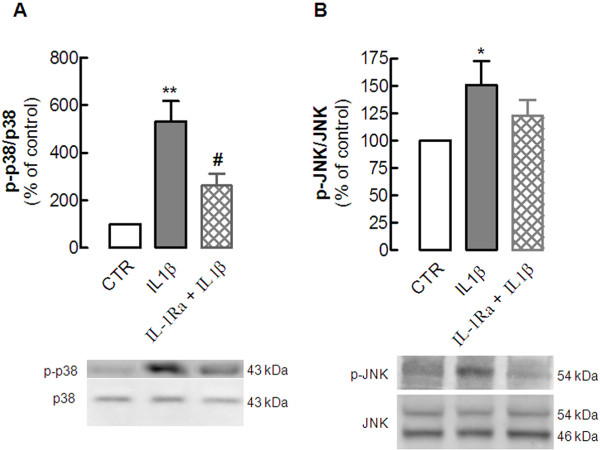
**Interleukin (IL)-1βtype Ireceptor mediatesthe IL-1β-inducedphosphorylation ofthe mitogen-activatedprotein kinases(MAPKs) Junkinase (JNK)and p38in hippocampalcultured neurons**. Rat hippocampal neuronal cultures at 7 days *in vitro* (DIV) were exposed to 100 ng/ml IL-1β for 15 minutes in the absence or in the presence of the antagonist of the IL-1β type I receptor, IL-1Ra (5 μg/ml), added 30 minutes before addition of IL-1β. Cells were then lysed on ice in a minimum volume of radio-immunoprecipitation assay (RIPA) buffer for western blotting analysis to quantify the immunoreactivity of the activated (phosphorylated) form of (**A**) p38 (p-p38) and (**B**) JNK (p-JNK). In both panels, the bar graphs display the mean ± SEM of three to four experiments, and the blots below illustrate a representative experiment showing that IL-1Ra (**A**) prevented the IL-1β-induced phosphorylation of p38 and (**B**) attenuated the phosphorylation of JNK. In each experiment, membranes were first used to detect each phosphorylated MAPK, and then reprobed for the total amount of MAPK, so that the activation of each MAPK was calculated as a ratio between the phosphorylated and total immunoreactivities, which were expressed as a percentage of control (CTR) values (that is, in the absence of any added drug). *P < 0.05 and **P < 0.01 compared with control; #*P* < 0.05 compared with the effect of IL-1β in the absence of IL-1Ra.

To evaluate the involvement of IL-1β type I receptors, we tested the effect of the endogenous antagonist IL-1Ra (5 μg/ml), which prevents the docking of the IL-1β receptor accessory protein to form the heterotrimeric complex that is necessary for signal transduction. Addition of 100 ng/ml IL-1β induced the phosphorylation of p38 (532.3 ± 85.5% of control; *P* < 0.01 versus control; n = 4) and JNK (151.0 ± 22.1% of control *P* < 0.05 versus control; n = 4) and IL-1Ra prevented this IL-1β-induced phosphorylation of p38 (261.3 ± 50.2% of control, *P* < 0.05 versus the absence of IL-1Ra; n = 4) and attenuated the activation (phosphorylation) of JNK (123.0 ± 20.0% of control, n = 3) (Figure 2). We did not test whether IL-1Ra affected the activation of MAPK.

### Synaptic and sub-synaptic localization of interleukin-1β type I receptor

**Figure 3:**
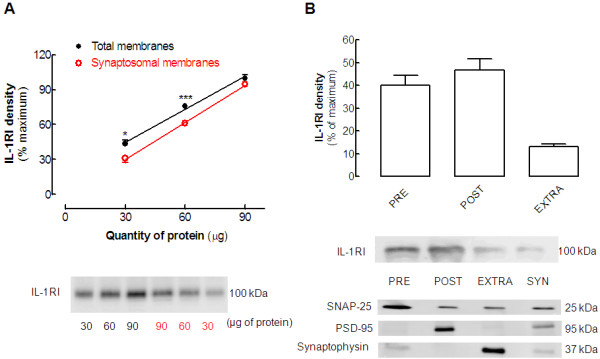
**Interleukin (IL)-1βtype 1receptor islocated innerve terminals,both inthe pre-synapticand post-synapticdensities.** (**A**) Comparison of the immunoreactivity of the IL-1β type 1 receptor (IL-1RI) in total membranes (comprising neuronal, glial, and endothelial membranes) and in membranes from purified nerve terminals (synaptosomes) prepared from the hippocampus of adult male Wistar rats. The graph displays the mean ± SEM of four experiments and the blot below illustrates a representative experiments, where we always used three different protein quantities (30, 60, and 90 μg) of both total and synaptic membranes of the same hippocampus in the same electrophoresis gel, and the percentage of IL-1RI immunoreactivity for each amount of protein was calculated relative to the maximum immunoreactivity obtained for each western blotting membrane (that is, that of the band obtained with 90 μg of total membranes). *P < 0.05 and ***P < 0.001 comparing the same quantities of total and synaptosomal membranes. (**B**) Sub-synaptic distribution of IL-1RI in a comparison of IL-1RI immunoreactivity in synaptosomal membranes (SYN) and in its fractions containing the pre-synaptic, post-synaptic, and extra-synaptic membranes, the purity of which was gauged by the segregation of the active zone marker synaptosomal-associated protein (SNAP)-25, the post-synaptic density (PSD) marker (PSD-95) and the extra-synaptic (vesicular) marker synaptophysin. The percentage of IL-1RI immunoreactivity was calculated relative to the maximum reactivity of each membrane and the data in the bar graph are mean ± SEM of n = 4 experiments.

Although a number of effects mediated by IL-1β receptor I have been reported to occur in brain cells [4,35], little is known about the localization of IL-1β type I receptor in neurons [13]. Thus, we investigated whether IL-1β type I receptors are indeed located in native brain neurons, paying particular attention to its putative synaptic and sub-synaptic localization. For this purpose, we first compared the density of IL-1β type I receptor immunoreactivity in total membranes and in synaptic membranes prepared from the hippocampus of adult rats. In all the western blots, the antibody used recognized a single well-defined band with an apparent molecular weight slightly below 100 kDa (higher than the expected molecular weight of 75 to 80 kDa expected for this protein). However, selectivity was not confirmed using genetic downregulation of the IL-1β type I receptor. With these limitations in mind, we found that the total membranes, which mostly comprise glial and endothelial cell membranes, were enriched with IL-1β type I receptor relative to the synaptic membranes (Figure 3A). For instance, with 30 μg of protein in the western blotting analysis, the total membrane portion displayed significantly higher (*P* < 0.05) IL-1β type I receptor immunoreactivity than the synaptosomal membrane portion (43.7 ± 3.2% compared with 31.1 ± 3.8% of total immunoreactivity, respectively, n = 4). This difference was also seen with 60 μg of protein (Figure 3A), but disappeared as the signal became saturated at 90 μg of protein (Figure 3A). Although the IL-1β type I receptor was mainly located outside synaptic regions, we further detailed its sub-synaptic distribution; the IL-1β type I receptor was located almost exclusively at post-synaptic (46.9 ± 4.8%) and pre-synaptic (40.0 ± 4.3%) sites. with a lower density at peri-synaptic sites (13.1 ± 1.3%, n = 3).

### The interleukin-1β-induced activation of mitogen-activated protein kinases is prevented by an A_2A_ receptor antagonist

**Figure 4:**
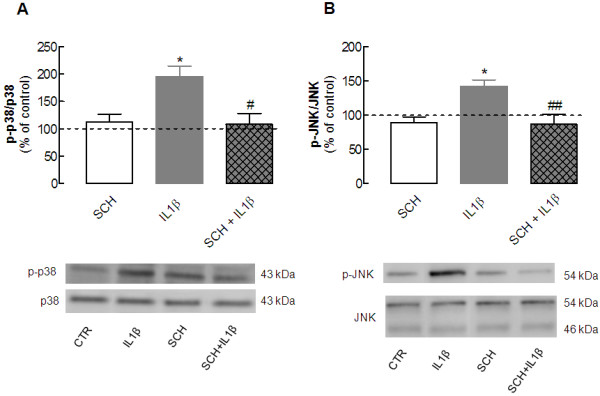
**Blockade ofadenosine A**_**2A**_**receptors preventsthe interleukin(IL)-1β-induced activationof themitogen-activated proteinkinases (MAPKs)Jun kinase(JNK) andp38 inhippocampal culturedneurons.** Rat hippocampal neuronal cultures at 7 days *in vitro* (DIV) were exposed to 100 ng/ml IL-1β for 15 minutes in the absence or presence of 50 nmol/l SCH58261, an antagonist of adenosine A_2A_ receptors, which was added 20 minutes before addition of IL-1β. Cells were then lysed on ice in a minimum volume of radio-immunoprecipitation assay (RIPA) buffer for western blotting analysis to quantify the immunoreactivity of the activated (phosphorylated) form of (**A**) p38 (p-p38) and (**B**) JNK (p-JNK). In both panels, the bar graphs display the mean ± SEM of five to six experiments, and the blots below illustrate a representative experiment showing that SCH58261 prevented the IL-1β-induced phosphorylation of (**A**) p38 and (**B**) JNK, without having any effect itself on either of these. In each experiment, membranes were first used to detect each phosphorylated MAPK and then reprobed for the total amount of MAPK, so that the activation of each MAPK was calculated as a ratio between the phosphorylated and total immunoreactivities, which were expressed as a percentage of control (CTR) values (that is*,* in the absence of any added drug). *P < 0.05 compared with control; #*P* < 0.05 and ##*P* < 0.01 compared with neurons exposed to IL-1β only.

The key question directing this study was to determine whether A_2A_R control the effect of IL-1β in neurons. For this purpose, we tested the ability of a previously validated A_2A_R antagonist, SCH58261, to prevent the IL-1β-induced activation of p38 and JNK in cultured neurons. Addition of 50 nmol/l SCH58261 20 minutes before exposing neurons to 100 ng/ml IL-1β for 15 minutes prevented the IL-1β-induced phosphorylation of p38 (108.0 ± 20.2% of control, compared with 195.5 ± 18.8% of control in the presence of IL-1β alone; *P* < 0.05; n = 6) (Figure 4A) and JNK (86.6 ± 14.6% of control, compared with 142.3 ± 9.1% of control in the presence of IL-1β alone; *P* < 0.01; n = 7) (Figure 4B), whereas SCH58261 alone failed to affect p38 or JNK phosphorylation (n = 6 to 7).

### Blockade of A_2A_ receptors prevents the interleukin-1β-induced exacerbation of neurotoxicity

**Figure 5:**
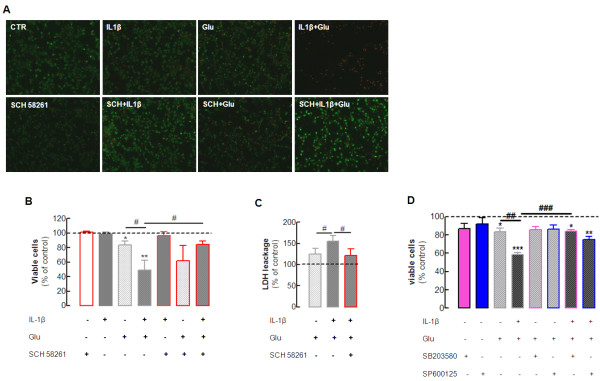
**Blockade ofadenosine A**_**2A**_**receptors preventsthe interleukin(IL)-1β-induced exacerbationof glutamate-mediatedexcitotoxicity inhippocampal culturedneurons.** Rat hippocampal neuronal cultures with 7 days *in vitro* (DIV) were exposed to vehicle (Krebs buffer) or to 100 ng/ml IL-1β , added 5 minutes before vehicle or 100 μmol/l glutamate (Glu) was added for 25 minutes, in the absence or in the presence of the selective A_2A_ receptor antagonist, SCH58261 (50 nmol/l), as indicated below each bar. Neurons were washed twice with Krebs buffer (pH 7.4) and fresh Neurobasal medium (with or without SCH58261) was added. Neurons were kept for 24 hours in the incubator (37°C, 95% O_2_/ 5% CO_2_) until analysis of neuronal viability using SYTO-13 plus propidium iodide (PI). (**A**) Representative images of SYTO-13 (green fluorescence) plus PI (red fluorescence) staining of nucleic acids for the indicated experimental conditions; (**B**) average results. (**C**) The significant findings were further confirmed using the lactate dehydrogenase (LDH) assay. Note that neither IL-1β nor SCH58261 alone had any effect on cell viability but IL-1β exacerbated Glu-induced neurotoxicity, which was abrogated by SCH58261. (**D**) The p38 inhibitor SB 203580 (10 μmol/l) and the JNK inhibitor SP600125 (10 μmol/l) respectively prevented and attenuated the exacerbation by IL-1β of glutamate-induced neurotoxicity, without affecting neuronal viability. Results are mean ± SEM of three to seven experiments (corresponding to the different cultures); *P < 0.05, **P < 0.01 and ***P < 0.001 compared with control (absence of any added drug), #*P* < 0.05, ##*P* < 0.01 and ###*P* < 0.001 compared between the indicated groups.

After establishing the key role of A_2A_R on the neuronal transduction pathways recruited by IL-1β, we next attempted to explore whether A_2A_R also controls the effect of IL-1β on neurotoxicity. Pro-inflammatory cytokines, particularly IL-1β, affect neurotoxicity by priming neurons to have increased susceptibility to neurotoxic insults [4,35]. We now investigated the effect of IL-1β on glutamate-induced neurodegeneration. This was achieved by incubating hippocampal neurons with 100 ng/ml IL-1β for 5 minutes before adding 100 μmol/l glutamate for 25 minutes, with evaluation of neuronal viability 24 hours later. This short time exposure to glutamate decreased neuronal viability by 21 ± 5% (*P* < 0.05 compared with control; n = 5) (Figure 5B), and IL-1β exacerbated this glutamate-induced neurotoxicity to 51 ± 13% (*P* < 0.05 compared with the effect of glutamate alone; n = 7), whereas IL-1β alone was devoid of effects on neuronal viability (99 ± 3% of viable cells, n = 6). Interestingly, 50 nmol/l SCH58261 prevented this exacerbation of glutamate-induced neurotoxicity caused by IL-1β (20 ± 5% loss of neuronal viability; *P* < 0.01 compared with the effect of glutamate in the presence of IL-1β; n = 5), whereas it failed to affect neurotoxicity significantly in the presence of glutamate alone (31 ± 17% loss of neuronal viability; n = 5). Finally, SCH58261 alone had no effect on neuronal viability (100 ± 2% of viable cells, respectively; n = 5).

We next confirmed this particular ability of A_2A_R to control the exacerbation of glutamate-induced neurotoxicity by IL-1β using another assay of neuronal damage, the release of LDH. Short-term exposure (25 minutes) to 100 μmol/l glutamate tended to increase LDH release by 25 ± 14% relative to the control (n = 6, *P* > 0.05) (Figure 5C) which was significantly (*P* < 0.05) potentiated in the presence of 100 ng/ml IL-1β (LDH release was 55 ± 13% above the control, n = 6). As previously shown with PI and SYTO-13, blockade of A_2A_R with 50 nmol/l SCH58261 abrogated the exacerbation of glutamate-induced neurotoxicity in the presence of IL-1β (LDH release was 21 ± 15% above control; *P* < 0.05 compared with the effect of glutamate in the presence of IL-1β; n = 6) (Figure 5C). Neither IL-1β nor SCH58261 alone significantly (*P* > 0.05) modified LDH release (data not shown).

Finally, in view of the combined evidence that A_2A_R prevented IL-1β-induced activation of p38 and JNK, and the exacerbation by IL-1β of glutamate-induced neurotoxicity, we next tested whether the inhibition of either p38 or JNK might also prevent the exacerbation by IL-1β of glutamate-induced neurotoxicity. Only the p38 inhibitor (SB203580, 10 μmol/l) effectively prevented the exacerbation by IL-1β of glutamate-induced neurotoxicity (As shown in Figure 5D), although the JNK inhibitor (SP600125, 10 μmol/l) also tended to ameliorate this effect, whereas neither of these inhibitors alone displayed any evident effect on neuronal viability.

### Blockade of A_2A_ receptors prevents the exacerbation caused by interleukin-1β of glutamate-induced calcium entry and calcium deregulation in cultured neurons

**Figure 6:**
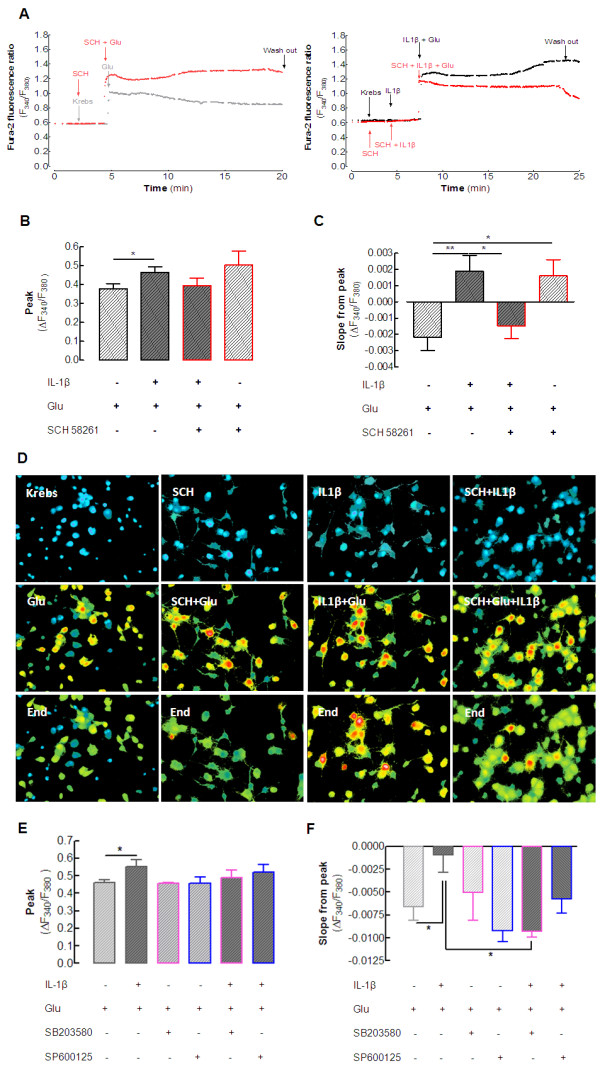
**Blockade ofadenosine A**_**2A**_**receptors preventsinterleukin (IL)-1β-inducedexacerbation ofglutamate-mediated calciumderegulation inhippocampal neurons.** Rat hippocampal neurons were loaded with the calcium probe Fura-2-AM, and the fluorescence emitted at 510 nm upon alternate excitation at 340 and 380 nm (F_340_ and F_380_, respectively) was monitored in cell bodies every second for 35 minutes. Basal fluorescence ratio was measured for the first 2 minutes, then drugs, prepared in normal Krebs solution, were added to the superfusion using a fast-pressurized system, as indicated by the arrows in (**A**). When used, 100 ng/ml IL-1β was added for 5 minutes before addition of 100 μmol/L glutamate (Glu). Cells were left in the presence of the stimuli for the next 15 minutes, and then washed through superfusion of normal Krebs. The A_2A_ receptor selective antagonist, SCH58261 (50 nmol/l), was incubated for 20 minutes before the beginning of the experiment and continued throughout. The results are presented as the ratio (F_340_/F_380_) of recordings of healthy neurons (evaluated by their response to 50 mmol/l KCl, after washing away the stimuli). IL-1β increased both (**B**) glutamate-induced calcium entry (peak value of the difference between the basal ratio and the stimuli-induced ratio) and (**C**) glutamate-induced calcium deregulation, calculated as the slope from peak values until the removal of the stimuli. (**B**) SCH58261 attenuated the exacerbation by IL-1β of glutamate-induced calcium and (**C**) prevented the calcium deregulation induced by the combination of IL-1β plus glutamate. However, SCH58261 seemed to aggravate the effect of glutamate alone. (**D**) Representative fluorescence images of the neuronal cell bodies (first row) in basal conditions, (middle row) in the presence of stimuli, and (bottom row) immediately before washing away the stimuli. The color scale of blue, green, yellow, red, and white corresponds to increasing calcium concentrations. (**E**) Ability of the p38 inhibitor (SB 203580, 10 μmol/L) to attenuate the exacerbation by IL-1β of glutamate-induced calcium entry, and (**F**) ability of both inhibitors to either attenuate (JNK inhibitor) or prevent (p38 inhibitor) calcium deregulation, whereas neither of the inhibitors by itself affected the dynamics of intracellular calcium. Data are mean ± SEM of four to six different cell cultures (480 to 1200 cells analyzed per condition for each n), *P < 0.05, **P < 0.01.

Previous studies have suggested that the effect of IL-1β on the priming of neuronal viability involves abnormal activation of NMDA receptor-mediated calcium influx [7,13,14]. Thus, we tested whether IL-1β could bolster the glutamate-induced calcium entry and calcium deregulation in neurons, and investigated the effect of A_2A_R blockade on these. We used a single-cell calcium imaging approach, loading hippocampal cultured neurons with the selective ratiometric calcium dye, Fura-2. We found that 100 μmol/l glutamate caused an immediate rise in intracellular free calcium concentration ([Ca^2+^_i_) as gauged by an increase in the Fura-2 fluorescence ratio of 0.38 ± 0.03 above the control (n = 6 cultures, 3 to 4 coverslips per culture, and 30 to 50 neurons analyzed per coverslip; *P* < 0.05). The presence of 100 ng/ml IL-1β consistently increased this effect of glutamate (ratio of 0.47 ± 0.03, n = 6 cultures, 3 to 4 coverslips per culture, and an average of 30 to 50 neurons analyzed per coverslip; *P* < 0,05) (Figure 6), whereas IL-1β alone failed to trigger any modification in the Fura-2 signal. Pre-incubation of cells with 50 nmol/l SCH58261 attenuated this effect of IL-1β on the glutamate-induced increase of [Ca2+]i (Fura-2 ratio of 0.40 ± 0.04; *P* < 0.05 compared with the effect of glutamate in the presence of IL-1β; n = 6 cultures, 3 to 4 coverslips per culture, and an average of 30 to 50 neurons analyzed per coverslip) (Figure 6). By contrast, SCH58261 actually tended to amplify the effect of glutamate alone (Fura-2 ratio of 0.50 ± 0.07, n = 4 cultures, 4 coverslips per culture, and an average of 30 to 50 neurons analyzed per coverslip) (Figure 6), whereas SCH58261 alone had no effect on [Ca^2+^_i_.

Apart from this initial effect of glutamate on calcium transients, we also evaluated how IL-1β and A_2A_R blockade affected the ability of neurons to adapt to the continuous presence of glutamate. Thus, we evaluated the variation of the Fura-2 fluorescence ratio from its peak value shortly after the addition of glutamate until the end of the incubation period with glutamate (that is, for 15 minutes). Most neurons were able to adapt to the continuous presence of glutamate and decrease their [Ca^2+^_i_ over time (slope of −0.0022 ± 0.0008, n = 6 cultures, 3 to 4 coverslips per culture, and an average of 30 to 50 neurons analyzed per coverslip) (Figure 6C). By contrast, in the presence of 100 ng/ml IL-1β, neurons lost their capacity to adapt to the continuous presence of glutamate, as testified by their tendency to continue increasing their [Ca^2+^i (slope of +0.0019 ± 0.0009; *P* < 0.01 compared with glutamate alone; n = 6 cultures, 3 to 4 coverslips per culture, and an average of 30 to 50 neurons analyzed per coverslip) (Figure 6C). Notably, blockade of A_2A_R with SCH58261 (50 nmol/l) inverted this effect of IL-1β (slope of −0.0015 ± 0.0008; *P* < 0.05 compared with the simultaneous presence of IL-1β and glutamate; n = 6 cultures, 3 to 4 coverslips per culture, and an average of 30 to 50 neurons analyzed per coverslip) (Figure 6). Again, SCH58261 selectively prevented the exacerbation by IL-1β of glutamate-induced responses, and in fact, SCH58261 actually enhanced the response to glutamate alone (slope of +0.0016 ± 0.001; *P* < 0.05 compared with glutamate alone; n = 4 cultures, 4 coverslips per culture, and an average of 30 to 50 neurons analyzed per coverslip) (Figure 6). This apparently contradictory ability of SCH58261 to increase slightly the glutamate-induced intracellular calcium dynamics and to abrogate the exacerbating effect of IL-1β on glutamate-induced effects probably results from the pleiotropic nature of A_2A_R-mediated signaling and its plasticity under different experimental conditions [18].

As a final attempt to link calcium deregulation upon exposure to glutamate and IL-1β with the A_2A_R-mediated control of the exacerbation by IL-1β of glutamate-induced neurotoxicity, we tested whether inhibition of either p38 or JNK might also prevent the exacerbation by IL-1β of the glutamate-induced dynamics of intracellular calcium in cultured neurons. The p38 inhibitor SB203580, (10 μmol/l) attenuated the exacerbation by IL-1β of glutamate-induced initial calcium entry (Figure 6E) and prevented the calcium deregulation (Figure 6F). The JNK inhibitor SP600125 (10 μmol/l) also attenuated the effect of IL-1β with glutamate, although this was not significant, and neither of these inhibitors alone displayed any evident effects. The striking parallel between the effects of SCH58261 and SB203580 is an additional finding suggesting that the blockade of A_2A_R is indeed selectively preventing the exacerbation by IL-1β of glutamate-induced calcium transients, although the pleiotropic nature of A_2A_R may mean there are additional effects of SCH58261 on glutamate-induced calcium transients in the absence of IL-1β.

## Discussion

In this study, we found that A_2A_R control the exacerbation of glutamate-induced excitotoxicity exerted by IL-1β; this effect mainly involves the control of the direct effect of IL-1β on neurons, as gauged by the prevention of IL-1β-induced activation of MAPKs and of IL-1β-induced exacerbation of glutamate-induced calcium deregulation and neuronal damage.

The first finding of this study is that IL-1β type I receptors are mainly localized at synaptic regions in the hippocampus of adult rats. The comparison of total membranes, which have a high content of glial and endothelial membranes, with membranes from purified nerve terminals (synaptosomes) showed that IL-1β type I receptors are indeed located in synapses, although they are more abundant in total membranes, in agreement with the well-established predominant expression and localization of IL-1β type I receptors in endothelial cells in the brain parenchyma [36,37]. However, IL-1β type I receptors have also been found to be expressed [38,39] and present [13] in neurons, especially in the context of brain diseases [40]. Our results are in agreement with the previously reported localization of IL-1β type I receptors at the PSD [13], as expected from the ability of IL-1β to control NMDA receptor-mediated currents both *in vitro*[7,14,41] and *in vivo*[43]. Additionally, we now report that IL-1β type I receptors are also present at the pre-synaptic active zone, as would be expected based on the ability of IL-1β to control the release of glutamate from nerve terminals [43]. Thus, IL-1β type I receptors do indeed seem to be present at synapses, precisely where glutamatergic receptors are more abundant and where glutamate-associated neurodegenerative processes are initiated.

This localization of IL-1β type I receptors in neurons, which has also been confirmed to occur in cultured hippocampal neurons [14], supports our observation that IL-1β can recruit various MAPKs in cultured neurons, in a manner sensitive to the inhibitor of IL-1β type I receptors, IL-1Ra. This agrees with previous reports that provided evidence indicating that certain MAPKs, particularly p38, play a crucial role in the mediating the physiopathological effects of IL-1β in the hippocampus [6,14,28]. Although phosphorylation of MAPKs can also promote neuroprotection under some conditions, the present study focused only on the potentially deleterious effects of IL-1β-induced phosphorylation of p38 and JNK. In fact, we found that this ability of IL-1β to recruit MAPKs, including p38, is by itself insufficient to trigger neuronal deregulation and damage. because IL-1β only primes neurons for enhanced susceptibility to neuronal damage, rather than itself directly triggering this damage [44]. We directly verified that IL-1β alone was indeed devoid of neuronal effects, but was able to potentiate glutamate-induced neurotoxicity in cultured hippocampal neurons, in agreement with the ability of IL-1β to exacerbate brain damage in conditions involving glutamate-induced excitotoxicity [7,8,35,42] and in agreement with the localization of IL-1β type I receptors in synapses, where ionotropic glutamate receptors are located. The present study adds a new mechanistic insight by showing that IL-1β causes a larger glutamate-induced entry of calcium into neurons and a late calcium deregulation upon exposure of cultured hippocampal neurons to glutamate. The later is of particular interest in view of the close association between late calcium deregulation and the irreversible loss of cellular, especially neuronal, viability [45]. This opens new avenues of research to explore the underlying mechanisms of this IL-1β-induced late calcium deregulation, which may be of key importance in the control of the inflammatory-mediated amplification loop mediating the propagation of brain damage [44].

As important as defining the mechanisms of inflammation-associated amplification of excitotoxic neuronal damage is the identification of novel strategies to control this mechanism, given its association with the evolution of brain damage. We found that the blockade of adenosine A_2A_R blunted the negative effect of IL-1β on neurons. This is of particular relevance in view of the ability of A_2A_R antagonists to prevent neuronal damage caused by various noxious brain insults [17,18,46]. This implies that these insults are able to trigger an increase in the extracellular levels of adenosine, which has already been reported to occur upon exposure to IL-1β [19,20]. We could not confirm this ability of IL-1β to trigger adenosine release under our experimental conditions, because the extracellular levels of adenosine in our cultured neurons were systematically below the detection limit of 100 nmol/l in the high-performance liquid chromatography method used [47]. Notably, A_2A_R blockade is effective both prophylactically and therapeutically [46]. Given that A_2A_R are enriched in cortical glutamatergic synapses [29], the prophylactic effect of A_2A_R antagonists is most probably related to the ability of A_2A_R to prevent synaptic dysfunction and damage, one of the early features of a number of brain disorders [48,49]. By contrast, the therapeutic beneficial effect of A_2A_R antagonists should depend on their ability to control a general feature associated with the amplification of brain damage, and neuroinflammation emerges as a potentially relevant candidate mechanism [17,18,23,46]. In line with this, we previously reported that A_2A_R antagonists prevent the induction of neuroinflammation [21,25]. This is now complemented by the demonstration that A_2A_R also controls the effect of a main pro-inflammatory cytokine, IL-1β, on neuronal viability. Thus, A_2A_R blockade displayed a particular ability to control the exacerbation of glutamate-induced neurodegeneration caused by IL-1β, extending the previous observation that A_2A_R blockade prevented the combined neurotoxicity of IL-1β and quinolinic acid [8]. Furthermore, our findings indicated the prime importance of the p38 MAPK as the transduction pathway associated with A_2A_R neuroprotection, as previously reported to occur in a number of noxious brain conditions [21,27,50]. Indeed, the striking parallel between the effects of SCH58261 and of the p38 inhibitor on the recovery of intra-neuronal calcium levels after the simultaneous exposure to glutamate and IL-1β supports our conclusion that A_2A_R play a key role in neuroinflammation-associated exacerbation of brain damage. In fact, both SCH58261 and SB203580 (the p38 inhibitor) were better at reverting the effect of IL-1β plus glutamate on calcium recovery than they were at changing the calcium peaks, which were only attenuated. Furthermore, the different results found for SCH58261 and SB203580 on the effect of glutamate alone on calcium intra-neuronal transients support our conclusion that A_2A_R have a dual role, preventing the exacerbation by IL-1β of glutamate-induced calcium dynamics and aggravating the direct effects of glutamate alone on calcium dynamics, consistent with the opposing roles of A_2A_R on inflammatory responses in the absence or presence of glutamate [25] derived from the well-known pleiotropic behavior of A_2A_R [18]. Clearly, the present results warrant further study into the potential role of A_2A_R in the control of glutamate-induced calcium deregulation. This is of particular interest because we have previously found that A_2A_R control mitochondria function [26,27], which plays a key role in the occurrence of calcium deregulation leading to neuronal damage [45] and is known to be involved in the etiology of diverse neurodegenerative disorders [51].

## Conclusion

The present study provides novel evidence indicating that A_2A_R control the signaling of IL-1β in neurons through p38, as well as the priming by IL-1β of glutamate-induced calcium entry and late calcium deregulation that are probably involved in the exacerbation of neuronal cell damage. Therefore, the present results prompt the hypothesis that the A_2A_R-mediated control of the priming effects of IL-1β might be a possible mechanism underlying the striking ability of A_2A_R antagonists to curtail neuronal damage caused by a variety of brain insults involving glutamate-induced neurotoxicity and neuroinflammation.

## Abbreviations

A2AR, A2A receptor; BSA, Bovine serum albumin; DTT, Dithiothreitol; EDTA, Ethylenediaminetetraacetic acid; ERK, Extracellular signal-regulated kinase; HEPES, 4-(2-hydroxyethyl)-1-piperazineethanesulfonic acid; IL-1β, Interleukin 1β; IL-1Ra, Interleukin-1β receptor antagonist; IL-1RI, interleukin-1β type 1 receptor; JNK, c-Jun N-terminal kinase; KHR, Krebs-HEPES-Ringer; LDH, Lactate dehydrogenase; MAPK, Mitogen-activated protein kinase; NAD+, Nicotinamide adenine dinucleotide; PAGE, Polyacrylamide gel electrophoresis; PBS, Phosphate-buffered saline; PMSF, Phenylmethanesulfonylfluoride; SCH58261, 2-(2-furanyl)-7-(2-phenylethyl)-7H-pyrazolo[4,3-e][1,2,4]triazolo[1,5-c]pyrimidin-5-amine; SDS, Sodium dodecyl sulfate; TBS-T, Tris-buffered saline with 0.1% Tween 20; Tris, Tris(hydroxymethyl)aminomethane.

## Competing interests

The authors report no conflict of interest.

## Authors’ contributions

AP Simões performed the neuronal cultures, the western blotting analyses, the calcium assays and the viability assays, and organized the manuscript. JA Duarte explored the synaptic and subynaptic localization of the IL-1β type 1 receptor. F Agasse provided some crucial initial assistance in the calcium assays. PM Canas worked on culturing neurons and on the viability assays. AR Tomé participated in the calcium assays and the HPLC determination of adenosine. PM Agostinho supervised all laboratory efforts. RA Cunha designed and coordinated the project. All authors read and approved the final manuscript.
